# Predictive Model of Acupuncture Adherence in Alzheimer Disease: Secondary Analysis of Randomized Controlled Trials

**DOI:** 10.2196/82787

**Published:** 2026-01-21

**Authors:** Ze-Hao Chen, Ran Li, Yu-Hang Jiang, Jia-Kai He, Shan-Shan Yan, Guan-Hua Zong, Zong-Xi Yi, Xin-Yu Ren, Bao-Hui Jia

**Affiliations:** 1 Department of Acupuncture and Moxibustion, Guang’anmen Hospital, China Academy of Chinese Medical Sciences, 5 Beixiange, Xicheng District, Beijing, China; 2 Department of Rehabilitation Medicine, Guang'anmen Hospital, China Academy of Chinese Medical Sciences, 5 Beixiange, Xicheng District, Beijing, 100053, China, 86 010 8800 1454; 3 Department of Traditional Chinese Medicine, Peking University People’s Hospital, Beijing, China; 4 Department of Acupuncture and Moxibustion, Guang'anmen Hospital, Beijing University of Chinese Medicine, Beijing, China

**Keywords:** alzheimer disease, acupuncture, adherence, nomogram, predictive model

## Abstract

**Background:**

The therapeutic efficacy of acupuncture in treating Alzheimer disease (AD) largely depends on consistent treatment adherence. Therefore, identifying key factors influencing adherence and developing targeted interventions are crucial for enhancing clinical outcomes.

**Objective:**

This study aims to develop and validate a predictive model for identifying patients with AD who are likely to maintain good adherence to acupuncture treatment.

**Methods:**

This secondary analysis included 108 patients with probable AD, aged 50 to 85 years, from 2 independent randomized controlled trials conducted at Guang’anmen Hospital, China Academy of Chinese Medical Sciences. Of all, 66 patients were assigned to the development cohort and 42 to the external validation cohort. Acupuncture adherence was defined as the proportion of completed sessions relative to scheduled sessions, with good adherence defined as ≥80% completion. Baseline data included demographic, clinical, cognitive, functional, psychological, and caregiving variables. Multivariable logistic regression with backward stepwise selection was used to identify significant predictors, and a nomogram was constructed based on the final model. Model performance was assessed using receiver operating characteristic curves, calibration plots, and decision curve analysis, with external validation performed by receiver operating characteristic analysis. Sensitivity analysis was performed using alternative adherence thresholds of 70% and 90%.

**Results:**

A higher number of treatments during the first month was associated with a significant increase in the odds of good adherence (odds ratio [OR] 3.06, 95% CI 1.68‐7.01; *P*=.002), while longer disease duration (OR 0.97, 95% CI 0.94‐1.00; *P*=.049) and receiving care from a part-time caregiver (OR 0.19, 95% CI 0.04‐0.72; *P*=.022) were associated with lower odds of adherence. Sensitivity analyses further supported the stability and reliability of the model.

**Conclusions:**

This study is the first to develop and validate a predictive model for acupuncture adherence in patients with AD. In clinical research, it can facilitate participant stratification and help identify individuals who may need additional adherence support, thereby reducing bias and enhancing trial quality. In clinical practice, the nomogram enables proactive adherence management by prospectively identifying high-risk patients and guiding targeted strategies to improve adherence and optimize therapeutic outcomes.

## Introduction

Alzheimer disease (AD) is a progressive neurodegenerative disorder primarily characterized by cognitive decline, functional impairment in activities of daily living, and neuropsychiatric symptoms [1]. Acupuncture, given its favorable safety profile and potential for symptomatic improvement [2-4], has been recommended as a promising nonpharmacological long-term therapy in clinical practice guidelines for the management of AD [5]. However, the therapeutic efficacy of acupuncture for AD remains a subject of ongoing debate [6,7]. This inconsistency in findings may be partially explained by a critical, yet often underinvestigated factor: treatment adherence.

Consistent adherence is crucial to therapeutic efficacy, particularly in chronic disease management and nonpharmacological interventions. At present, adherence has been studied primarily in the context of pharmacotherapy, with reported rates of long-term pharmacological adherence in patients with AD ranging from 16.5% to 51% [8,9]. Some studies have developed models to predict patients’ medication adherence [10,11]. In contrast, adherence to nonpharmacological therapies such as acupuncture remains insufficiently explored. This knowledge gap is compounded by the lack of a standardized definition for what constitutes good or poor adherence. This variability in definitions across studies makes it difficult to compare findings and may be a key confounding factor obscuring the true dose-response relationship of acupuncture [12].

Clinical studies have shown that factors influencing acupuncture adherence are multifaceted, encompassing patient subjective norms, perceived behavioral control, and treatment commitment [13]. Specific factors include perceived effectiveness, family support, emotional status, patient recognition and acceptance of acupuncture, and the availability of medical subsidies [14]. Nonetheless, most of these studies have provided only descriptive insights or general recommendations based on literature reviews [15,16], while few have offered quantitative evidence derived from clinical data. Notably, research specifically focusing on acupuncture adherence in individuals with AD is exceedingly scarce. Given the substantial cognitive decline, behavioral symptoms, and caregiver dependence associated with AD, patients in this population may face unique barriers to maintaining adherence. Thus, findings from studies in other populations may not be directly generalizable to patients with AD. Understanding the specific determinants of adherence in this unique population is crucial for optimizing treatment delivery and improving outcomes.

Given the ongoing debate over acupuncture’s efficacy and the critical yet poorly understood role of adherence in treatment outcomes, identifying predictors for treatment engagement in a methodologically robust setting is paramount. In this study, we conducted a secondary analysis of data from 2 independent randomized controlled trials (RCTs) previously conducted by our team, with the aim of identifying key predictors of acupuncture adherence among patients with AD and developing a clinically applicable predictive model. By identifying individuals at high risk of poor adherence and enabling the development of targeted intervention strategies, this study seeks to enhance the adherence and efficacy of acupuncture treatment and provide evidence-based support for adherence management in nonpharmacological interventions for AD.

## Methods

### Study Design and Data Source

This study was a secondary analysis based on data collected from 2 independent RCTs conducted at Guang’anmen Hospital, China Academy of Chinese Medical Sciences (CACMS). Participants were primarily recruited between December 2021 and June 2024. The first RCT, conducted from December 2021 to June 2024, enrolled 66 patients and served as the model development cohort. The second RCT, conducted from June 2022 to November 2022, included 42 patients and served as an external validation cohort for the predictive model.

Eligibility screening and clinical assessments were conducted by licensed physicians from the departments of encephalopathy and neurology. Inclusion criteria and exclusion criteria are present in Textbox 1.

Textbox 1. **Inclusion and exclusion criteria.**

**Inclusion criteria:**
  - A diagnosis of probable Alzheimer disease according to the National Institute on Aging–Alzheimer’s Association criteria [17]
  - Age between 50 and 85 years
  - A Clinical Dementia Rating score ≥0.5
  - A Mini-Mental State Examination score ≤26
  - A Hachinski Ischemic Scale score ≤4

**Exclusion criteria:**
  - Other neurological or systemic disorders known to cause progressive cognitive impairment
  - Recent use of medications or exposure to substances known to impair cognition
  - A history of trypanophobia or active skin infections
  - Acupuncture or electroacupuncture treatment within the past 2 weeks
  - Participation in other clinical trials

### Ethical Considerations

Both studies were conducted in accordance with the ethical principles outlined in the Declaration of Helsinki. This study is a secondary analysis of data derived from 2 RCTs previously conducted at Guang’anmen Hospital, CACMS. Both original trials received independent ethical approval from the ethics committee of Guang’anmen Hospital, CACMS (approval: 2021-056-KY-01 and 2022-087-KY). Written informed consent was obtained from all participants or their legally authorized representatives prior to data collection. Participants did not receive compensation for participation in the original trials. All treatments and assessments were provided free of charge. To protect participant privacy, all data used in this secondary analysis were anonymized and deidentified prior to analysis.

### Treatment and Adherence Assessment

Participants received 20-minute acupuncture sessions 3 times per week (on nonconsecutive days) for a total of 12 weeks. Adherence was assessed by calculating the proportion of completed treatment sessions relative to the total number of scheduled sessions during the intervention period. The proportion of days covered (PDC) was used as the adherence metric. In the absence of an established gold standard for adherence in nonpharmacological trials like acupuncture, we adopted the widely accepted threshold of PDC ≥80% from pharmacotherapy research [9]. This threshold is a well-validated proxy for sufficient exposure to treatment in chronic disease management. Participants with a PDC ≥80% were classified as having good adherence, while those with a PDC <80% were categorized as having poor adherence.

### Data Collection

The following clinical data were collected: sex, age, disease duration, disease severity, educational level, occupation, history of acupuncture treatment, Mini-Mental State Examination score, Alzheimer’s Disease Assessment Scale–Cognitive Subscale score, basic activities of daily living, instrumental activities of daily living, presence of behavioral and psychological symptoms of dementia, Patient Health Questionnaire-9 score for depressive symptoms, caregiving role, number of treatments in the first month, travel time to the hospital, recruitment method, and adherence outcomes.

### Screening of Influencing Factors

A linear regression model was used to assess multicollinearity among independent variables. Multicollinearity was quantified by calculating the variance inflation factor and tolerance values. Variables with a variance inflation factor <10 and tolerance >0.1 were considered to have acceptable levels of multicollinearity [18]. Variables meeting these criteria were subsequently included in a multivariable logistic regression model. Variable selection was performed using a backward stepwise regression approach based on the likelihood ratio test, with a significance threshold of *P*<.05 for retention in the model. This data-driven approach was chosen to build a parsimonious model and reduce the risk of overfitting. We also conducted exploratory analyses by forcing clinically relevant but nonsignificant variables into the model, but this did not lead to a significant improvement in model performance and increased model complexity. Therefore, the final model retained only the statistically significant predictors. Final variables were required to meet criteria for statistical significance, low multicollinearity, and satisfactory predictive performance. To improve model interpretability, Shapley Additive Explanations (SHAP) values were calculated to quantify the relative contribution of each predictor to the outcome [19]. SHAP values were visualized using beeswarm plots.

### Construction and Evaluation of the Nomogram

A nomogram was developed based on the final multivariable logistic regression model to predict adherence. The nomogram serves as a graphical tool to visualize the relationships between multiple predictors and the outcome, facilitating individualized risk assessment and clinical decision-making. In the nomogram, each predictor is aligned with its corresponding axis; by drawing a vertical line from the predictor’s value to the point scale, a score can be assigned. The total score, obtained by summing the individual scores, corresponds to a predicted probability of adherence on the nomogram’s outcome axis.

Model performance was assessed through receiver operating characteristic (ROC) curve analysis, calibration plots, the Hosmer-Lemeshow goodness-of-fit test, and decision curve analysis using the development dataset. Internal validation was conducted using the bootstrap method with 1000 resampling iterations. Agreement between predicted and observed outcomes was evaluated using the κ statistic. An area under the ROC curve (AUC) between 0.5 and 0.7 was interpreted as indicating low discrimination, 0.7 to 0.9 as moderate, and >0.9 as high discrimination [20]. The nomogram’s robustness was further evaluated by performing ROC analysis in the external validation cohort.

### Statistical Methods

Data completeness for the variables included in the final model was assessed prior to analysis. There were no missing values for the variables included in the final model in either the development or validation cohorts, as complete data collection was a requirement for the per-protocol analysis in the 2 RCTs. All statistical analyses were conducted using R software (version 4.4.2; R Foundation for Statistical Computing). Continuous variables with a normal distribution were presented as mean (SD), while nonnormally distributed variables were expressed as median (IQR), with distribution assessed using the Shapiro-Wilk test. Categorical variables were reported as frequencies and percentages. Group comparisons were performed using independent-samples *t* tests for normally distributed continuous variables and the Mann-Whitney *U* test for nonnormally distributed continuous variables. Categorical variables were compared using the chi-square test or the Fisher exact test, as appropriate. All statistical tests were 2-tailed, and a *P* value <.05 was considered statistically significant. To assess the robustness of our model to the primary adherence definition (PDC ≥80%), a sensitivity analysis was performed by repeating the multivariable logistic regression using alternative thresholds of 70% and 90%.

## Results

### Baseline Characteristics of the Training Set

The predictive model was developed using data from the development cohort (cohort 1), which comprised 66 patients with AD (Table 1). Participants were stratified by treatment adherence into a good adherence group (n=43) and a poor adherence group (n=23). Of the total participants, 34 (51.5%) were female participants and 32 (48.5%) were male participants. The mean age was 71.8 (SD 7.9) years, and the mean disease duration was 50.0 (SD 26.0) months. Univariate analysis revealed significant differences between the good and poor adherence groups in caregiving status (*P*=.025) and the number of treatment sessions during the first month (*P*=.001).

The *P* values for testing differences between patients with good and poor adherence to acupuncture treatment were derived from independent samples *t* tests, Mann-Whitney *U* tests and chi-square tests or the Fisher exact tests.

**Table 1.** Comparison of baseline characteristics between patients with good and poor adherence (n=66).

Variable	Total	Good adherence (n=43)	Poor adherence (n=23)	*t* test (*df*)^a^	Wilcoxon rank-sum test^a^	Chi-square (*df*)	*P* value
Sex, n (%)				—	—^b^	1.88 (1)	.171
Female	34 (52)	19 (44)	15 (65)				
Male	32 (48)	24 (56)	8 (35)				
Age (y), mean (SD)	71.8 (7.9)	70.9 (7.7)	73.5 (8.3)	−1.28 (64)	—	—	.204
Disease duration (mon), mean (SD)	50.0 (26.0)	48.1 (26.0)	53.5 (26.2)	−0.80 (64)	—	—	.427
Disease severity,^c^ n (%)				—	—	—	.490
Mild	29 (44)	21 (49)	8 (35)				
Moderate	28 (42)	16 (37)	12 (52)				
Severe	9 (14)	6 (14)	3 (13)				
Education level, n (%)				—	—	0.05 (1)	.816
No higher education	40 (61)	27 (63)	13 (57)				
Higher education	26 (39)	16 (37)	10 (43)				
Occupation, n (%)				—	—	0.00 (1)	.99
Manual work	21 (32)	14 (33)	7 (30)				
Nonmanual work	45 (68)	29 (67)	16 (70)				
MMSE^d^, median (IQR)	16.5 (11.0‐21.0)	18.0 (12.0‐21.5)	16.0 (9.5‐20.0)	—	582	—	.241
ADAS-Cog^e^, median (IQR)	22.0 (15.0‐38.8)	22.0 (14.5‐39.0)	24.0 (16.0‐38.5)	—	454	—	.590
BADL^f^, median (IQR)	10.0 (9.0‐13.0)	10.0 (9.0‐12.5)	11.0 (9.5‐13.5)	—	444.5	—	.500
IADL^g^, median (IQR)	26.0 (18.2‐33.0)	27.0 (16.0‐32.5)	25.0 (19.5‐33.5)	—	472.5	—	.772
BPSD^h^,^c^ n (%)				—	—	—	.99
Present	53 (80)	34 (79)	19 (83)				
Absent	13 (20)	9 (21)	4 (17)				
PHQ-9^i^,^c^ n (%)				—	—	—	.99
Depressive symptoms	3 (5)	2 (5)	1 (4)				
Normal	63 (95)	41 (95)	22 (96)				
Travel time to the hospital (min), median (IQR)	60.0 (40.0‐100.0)	50.0 (35.0‐95.0)	60.0 (50.0‐135.0)	—	413	—	.272
First-month treatment sessions, median (IQR)	11.0 (11.0‐12.0)	12.0 (11.0‐12.0)	10.0 (8.0‐12.0)	—	736	—	.001
Caregiving role, n (%)				—	—	5.05 (1)	.025
Full-time caregiver	34 (52)	27 (63)	7 (30)				
Part-time caregiver	32 (48)	16 (37)	16 (70)				
History of acupuncture, n (%)				—	—	0.00 (1)	.99
No	30 (45)	20 (47)	10 (43)				
Yes	36 (55)	23 (53)	13 (57)				
Recruitment method^c^, n (%)				—	—	—	.844
Nursing home	4 (6)	3 (7)	1 (4)				
Multimedia	30 (45)	19 (44)	11 (48)				
Study team referral	15 (23)	11 (26)	4 (18)				
Outpatient clinic	17 (26)	10 (23)	7 (30)				

a Continuous variables were compared using independent-samples *t* tests when normally distributed and Wilcoxon rank-sum tests otherwise. Categorical variables were compared using the chi-square test or Fisher exact test, as appropriate.

b —: not applicable.

c Evaluated using the Fisher exact test.

d MMSE: Mini-Mental State Examination.

e ADAS-Cog: Alzheimer’s Disease Assessment Scale–Cognitive Subscale.

f BADL: basic activities of daily living.

g IADL: instrumental activities of daily living.

h BPSD: behavioral and psychological symptoms of dementia.

i PHQ-9: Patient Health Questionnaire-9.

### Independent Predictors of Adherence to Acupuncture Treatment Among Patients with AD

**Figure 1.**
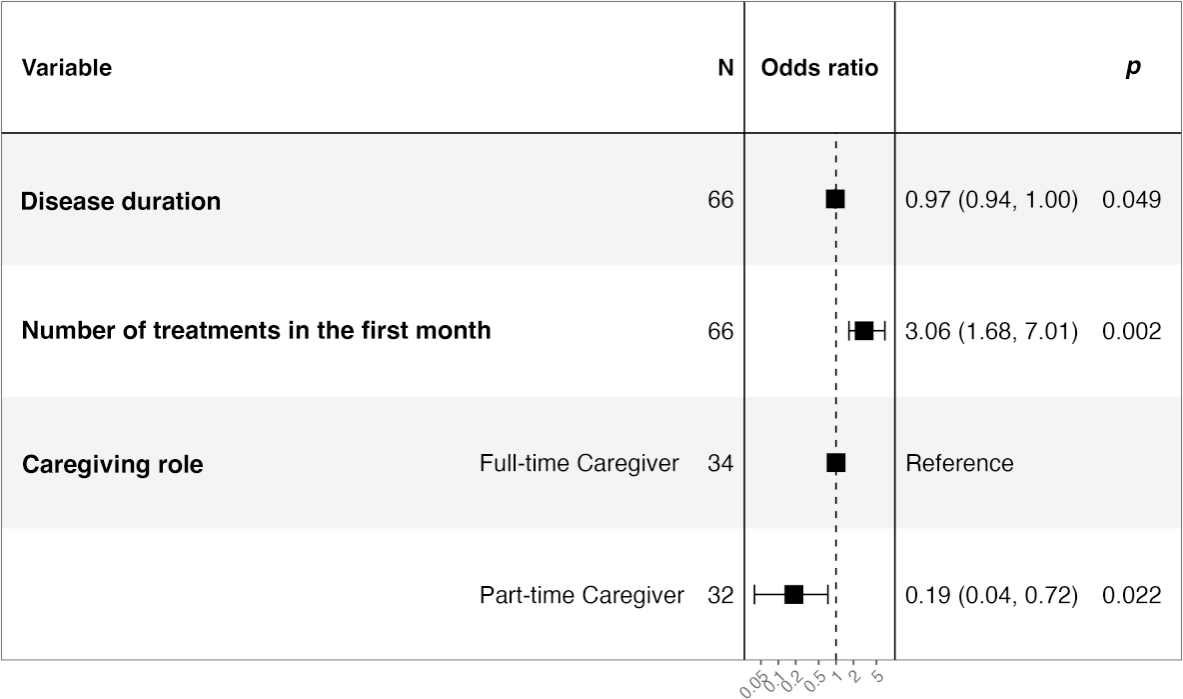
Forest plot of factors influencing adherence to acupuncture treatment among patients with Alzheimer disease based on multivariable logistic regression analysis. Dots represent odds ratios, and horizontal lines indicate 95% CIs. Longer disease duration and care provided by part-time caregivers were associated with lower adherence (odds ratio<1), while a higher number of treatment sessions during the first month significantly increased adherence (odds ratio >1). All variables included in the model were statistically significant (*P*<.05).

Initially, 17 potential predictors of adherence to acupuncture treatment in patients with AD were considered. Following multicollinearity analysis and assessment of clinical relevance, 16 variables were ultimately selected for model construction (Table S1 in Multimedia Appendix 1). Multivariable logistic regression analysis was performed to develop a predictive model of adherence to acupuncture treatment in this population. The final model is represented by the following equation (Figure 1; Table S2 in Multimedia Appendix 1):


$$\begin{array}{l} log\left(\frac{p}{1-p}\right)=-9.244-0.028\times \text{Disease}\text{Duration}+1.120\times \\ Number\;of\;Treatments\;in\;First\;Month-1.683\times \text{Caregiving}\;\text{Role} \end{array}$$

**Figure 2.**
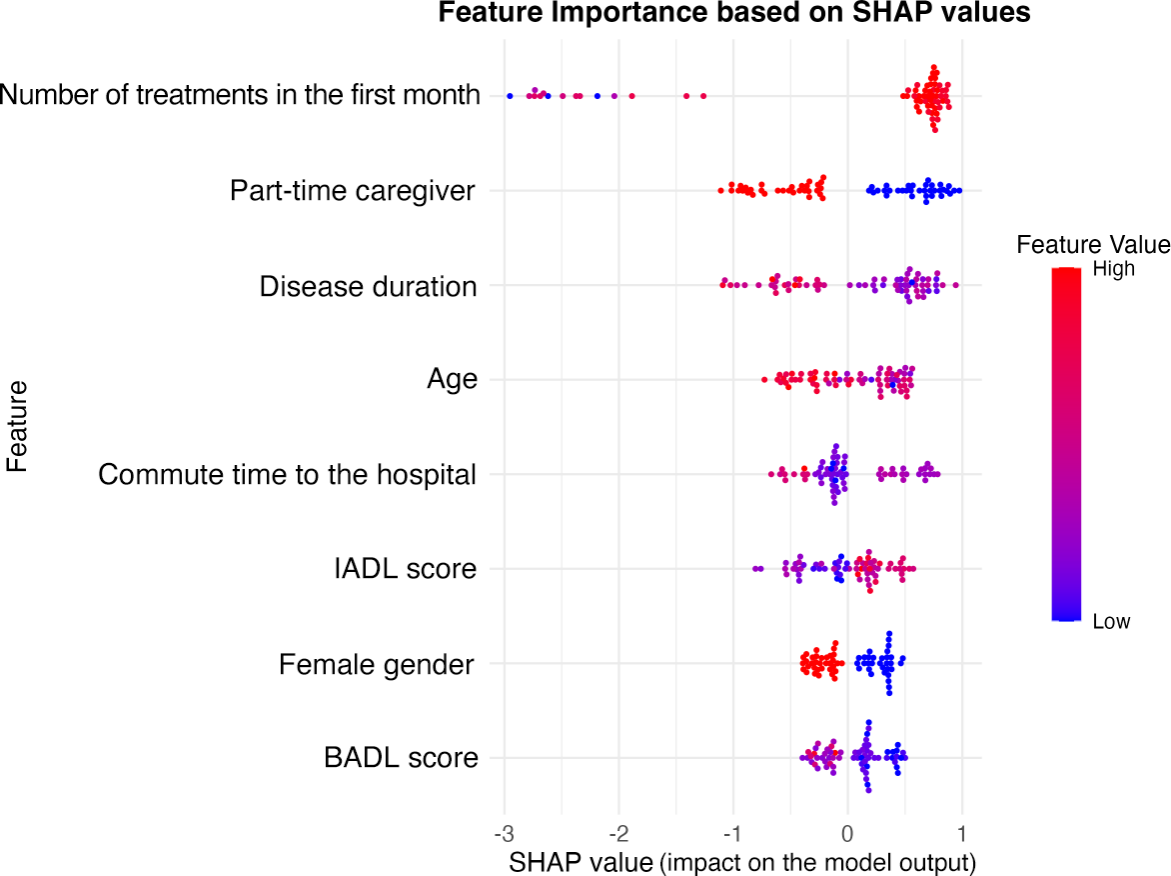
Shapely Additive Explanations (SHAP) summary plot of variable importance for predicting adherence to acupuncture treatment in patients with Alzheimer disease based on multivariable logistic regression analysis. BADL: basic activities of daily living; IADL: instrumental activities of daily living.

Disease duration, the number of treatments in the first month, and caregiving role were independent predictors of adherence to acupuncture treatment among patients with AD. A higher number of treatments during the first month was associated with a significant increase in the odds of good adherence (odds ratio [OR] 3.06, 95% CI 1.68‐7.01; *P*=.002), while longer disease duration (OR 0.97, 95% CI 0.94‐1.00; *P*=.049) and receiving care from a part-time caregiver (OR 0.19, 95% CI 0.04‐0.72; *P*=.022) were associated with lower odds of adherence. SHAP analysis quantified the contributions of these predictors, confirming their importance in the model (Figure 2). The SHAP results were consistent with the logistic regression findings, enhancing the interpretability of the model.

The results indicated that the number of treatment sessions during the first month, caregiving role, and disease duration were the 3 most important factors influencing adherence. This ranking was highly consistent with the findings of the logistic regression model, demonstrating the substantial contribution of these variables to the model’s predictive performance.

The SHAP summary plot visualizes the impact of each variable on the predicted probability of adherence. Each dot represents the SHAP value of an individual observation, indicating the degree and direction of that variable’s influence on the model output. Higher SHAP values correspond to a stronger positive contribution to adherence probability. The color gradient from blue to red indicates the relative value of each variable for that observation (blue =lower value; red =higher value). For example, a higher number of treatments in the first month (red) is associated with higher SHAP values, reflecting its positive impact on adherence.

### Development and Evaluation of the Nomogram

**Figure 3.**
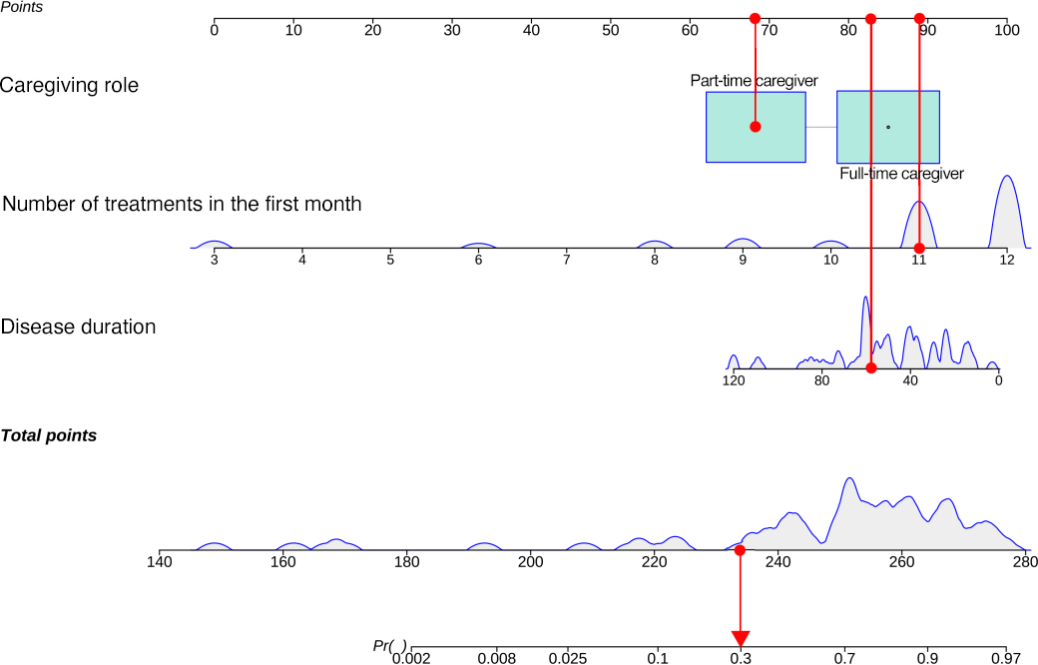
Nomogram for predicting adherence to acupuncture treatment among patients with Alzheimer disease.

**Figure 4.**
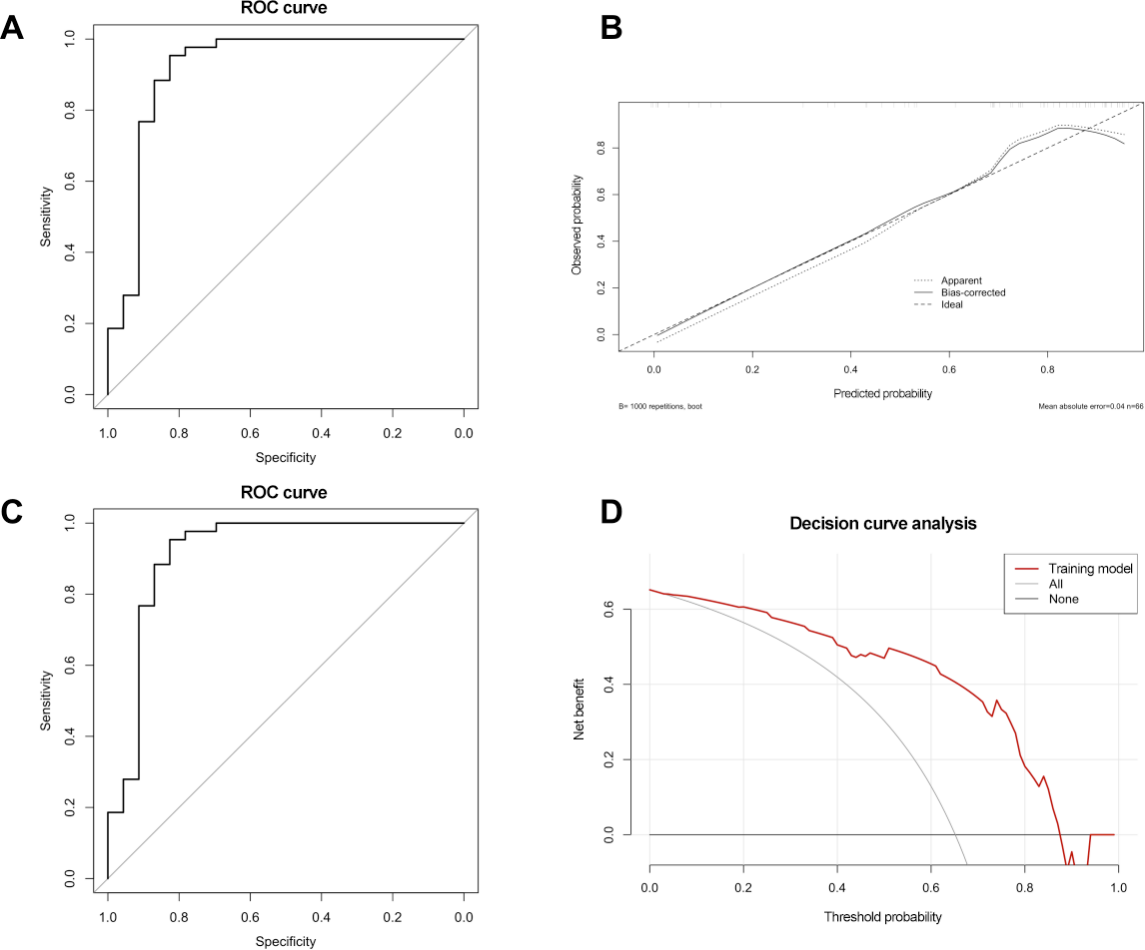
Evaluation and validation of the nomogram model for predicting adherence to acupuncture treatment in patients with Alzheimer disease. (A) Receiver operating characteristic (ROC) curve assessing the discriminative ability of the model, with an area under the ROC curve of 0.914, indicating excellent predictive performance. (B) Calibration curve illustrating agreement between predicted and observed adherence probabilities. The x-axis represents predicted probability, while the y-axis shows the observed probability of adherence. The Ideal line indicates perfect concordance between predicted and actual outcomes. The apparent line reflects model performance in the original sample, and the bias-corrected line shows model performance adjusted for overfitting using the bootstrap method (1000 resamples). The close alignment of the calibration curves with the ideal line demonstrates the high accuracy and reliability of the model. (C) ROC curve of the nomogram model in the validation cohort, with an area under the ROC curve of 0.838. (D) Decision curve analysis of the nomogram model in the training cohort. The x-axis represents the threshold probability, and the y-axis indicates the net clinical benefit across different thresholds.

A nomogram was developed based on the final predictive model, incorporating the number of treatments in the first month, disease duration, and caregiving role (Figure 3). The model demonstrated excellent discrimination, with an area under the ROC curve (AUC) of 0.914 (Figure 4A). Validation was performed using cohort 2 (test set), which included 42 patients with AD (Table S3 in Multimedia Appendix 1). The calibration curve from internal validation indicated good agreement between predicted and observed probabilities (mean absolute error =0.04; mean squared error =0.003; 90th quantile absolute error =0.078) (Figure 4B). The Hosmer-Lemeshow goodness-of-fit test showed that the model predictions were well calibrated (*χ*²_8_=10.9; *P*=0.21). The decision curve analysis showed that the nomogram provided a net clinical benefit across a wide range of threshold probabilities (Figure 4D). Internal validation yielded an overall predictive accuracy of 89.4%, with a κ statistic of 0.759, indicating good consistency and potential clinical utility. Furthermore, the model performed well in the external validation cohort, achieving an AUC of 0.833 (Figure 4C).

For example, a patient with AD with a disease duration of 60 months, part-time caregiving, and 11 acupuncture sessions during the first month would obtain corresponding point values on each variable axis, indicated by red dots on the upper horizontal scales. The points for the 3 variables are summed to yield a total score of approximately 234. On the bottom axis, a total score of 234 corresponds to a predicted adherence probability of approximately 0.30, as marked by the red arrow. This relatively low predicted probability highlights the need for proactive adherence management and targeted interventions in clinical practice.

### Sensitivity Analysis

To evaluate the robustness of our model, a sensitivity analysis was performed using alternative adherence thresholds of 70% and 90%. The model’s predictive performance remained strong across all definitions, with an area under the ROC curve (AUC) of 0.892 for the 70% threshold and 0.860 for the 90% threshold. The statistical significance of the number of treatments in the first month (*P*=.01 at 70%; *P*=.05 at 90%), caregiving role (*P*=.03 at 90%), and disease duration (*P*=.08 at 70%; *P*=.09 at 90%) fluctuated near the *P*=.05 cutoff; this could be caused by the dataset size. Overall, these findings support the stability of our model. Detailed results of the sensitivity analysis are provided in the supplementary materials (Figure S1 in Multimedia Appendix 1).

### Adverse Events

Safety monitoring was conducted throughout both RCTs. No serious adverse events related to acupuncture treatment were reported in either the development cohort or the validation cohort. Adverse events unrelated to the intervention are summarized in Table S4 in Multimedia Appendix 1.

## Discussion

### Predictors of Treatment Adherence in Patients with AD

This study identified the number of treatments in the first month, caregiving role, and disease duration as significant predictors of adherence to acupuncture treatment among patients with AD. The predictive nomogram constructed with these 3 variables demonstrated excellent discrimination in the development cohort (AUC=0.914) and acceptable performance in the external validation cohort (AUC=0.838). With its simplicity, interpretability, and ease of application, the nomogram may serve as a practical tool to assist clinicians in identifying patients at risk of poor adherence and tailor intervention strategies accordingly.

To understand the clinical implications of these predictors, it is useful to interpret them through the established framework of intentional versus unintentional nonadherence. Unintentional nonadherence typically arises from practical barriers, which can be internal to the patient’s condition (eg, forgetfulness and functional limitations) or external and situational (eg, transportation difficulties, inclement weather, and systemic disruptions like a pandemic). In contrast, intentional nonadherence involves a deliberate decision to cancel the treatment plan, often driven by subjective factors such as a perceived lack of efficacy, treatment fatigue, or shifting personal priorities. Our findings suggest that the identified predictors likely influence adherence through mechanisms related to both categories.

### Increasing the Number of Treatments in the First Month May Improve Adherence

A key finding of this study is that a higher number of treatments during the first month was a powerful predictor of adherence. This suggests that early and intensive engagement is critical for establishing sustained treatment behaviors. From the perspective of unintentional nonadherence, a structured, frequent schedule in the initial phase may help patients and caregivers with cognitive and organizational deficits to more quickly accept the therapeutic routine, making it a habitual part of their lives [21].

This intensive approach can also mitigate intentional nonadherence through two possible ways. First, given that the therapeutic effect of acupuncture is often cumulative [22], increasing treatment frequency may accelerate the perception of clinical benefits [23,24]. When patients and caregivers observe an improvement early on, their motivation and belief in the treatment’s value are naturally reinforced [25]. Second, frequent sessions offer more opportunities for communication among patients, caregivers, and clinicians, enabling the early detection and management of emerging issues. However, this approach requires careful consideration. It is important to acknowledge that a higher treatment frequency does not universally guarantee improved clinical outcomes [26] and may increase the treatment burden on families. The optimal number and timing of sessions may vary depending on disease severity, stage, and individual patient needs [27]. In conclusion, tailoring the intensity of acupuncture interventions to individual profiles remains a key consideration for future research and clinical practice for patients with AD, balancing clinical benefit and treatment burden.

### Caregiver Capacity Is a Critical Determinant of Adherence

Our finding that patients supported by part-time caregivers (defined as providing fewer than 41 h of care per week [28]) were significantly less likely to adhere underscores the critical role of caregiver capacity in treatment engagement. This highlights a powerful driver of unintentional nonadherence, as dementia caregiving is a long-term, high-burden undertaking [29-31]. In advanced stages, care demands can often exceed 100 hours per week [32]. This presents formidable logistical barriers for part-time caregivers, who are typically adult children trying to balance employment, their own household duties, and the challenge of not living with the person they care for. These challenges frequently limit their ability to schedule and accompany patients to appointments, provide consistent emotional support, or respond to emergent care needs, thereby compromising adherence despite their best intentions [29].

This relentless demand also takes a significant physical and psychological toll, leading to caregiver burden and fatigue that can further diminish the capacity to support treatment [33-37]. These inherent difficulties are often compounded by unpredictable external factors, such as sudden illness or bad weather, which can disproportionately disrupt the routines of caregivers with less flexibility. A more subtle yet powerful factor is a form of intentional nonadherence driven by altruism, where patients may forgo appointments out of a desire not to burden their children, ultimately leading to treatment discontinuation.

In contrast, spouses often serve as full-time caregivers (≥41 h/wk) [38,39] and are typically more emotionally invested and committed to maintaining treatment routines. Some may even retire early to provide round-the-clock care [40]. Our study also found that professional caregiving within institutional settings can offer stable, structured, and high-quality care, which may facilitate better adherence. To enhance adherence in clinical practice, health care providers should actively involve caregivers in treatment planning and offer tailored education to improve their understanding of disease progression, treatment goals, and the importance of adherence [41,42]. Such interventions can mitigate both unintentional nonadherence (by improving scheduling and problem-solving skills) and intentional nonadherence (by reinforcing the perceived value of the treatment). Strengthening caregivers’ motivation and capacity to support treatment is essential. When informal caregiving resources are insufficient, incorporating professional home care services or transitioning to institutional care may help ensure treatment continuity and effectiveness. Notably, countries such as Denmark and the Netherlands have developed comprehensive formal care systems for dementia that integrate medical and social support services [30,43]. These models may provide valuable reference points for improving dementia care infrastructure in China.

### Early Detection, Prevention, and Intervention Still Key to Treating AD

Our study found that longer disease duration was associated with poorer adherence to acupuncture treatment. Primarily, this is a form of unintentional nonadherence driven by the patient’s own progressive cognitive and functional decline, which impairs their capacity to independently manage appointments [44]. A higher prevalence of neuropsychiatric symptoms can reduce patient cooperation, while accumulating physical comorbidities and mobility limitations create new logistical hurdles. As the disease advances, a cascade of factors converges to further undermine adherence. This decline simultaneously increases the burden on caregivers, who may experience emotional exhaustion and a deterioration in their own health, diminishing their ability to provide consistent support [45]. The combination of increasing patient dependency, rising caregiver exhaustion, and mounting logistical obstacles creates a formidable barrier to sustained treatment in the later stages of the disease.

This underscores the critical importance of a proactive and early approach to management. Our findings emphasize that the timing of intervention is paramount. Initiating treatment when patients retain greater cognitive and functional capacity offers a crucial window of opportunity. Early initiation of acupuncture may help establish regular treatment routines, foster therapeutic rapport, and enhance patient motivation. Moreover, health care providers should prioritize early education and ongoing support for both patients and caregivers. This includes training in caregiving skills, psychological counseling, and practical strategies to reduce caregiver burden and enhance quality of life [46]. These factors can contribute not only to improved adherence but also to more favorable long-term outcomes by potentially slowing the trajectory of disease progression.

### Limitations

This study has limitations. First, although this study suggests that early, intensive treatment may enhance adherence, the optimal frequency and total number of acupuncture sessions for patients with AD remain undetermined. In real-world settings, increasing treatment frequency may impose greater transportation, time, and financial burdens on both patients and caregivers, potentially reducing their motivation and adherence. Second, our analysis was based on data from previous RCTs that were provided free of charge. Consequently, we could not assess the influence of crucial socioeconomic factors, such as treatment costs, or household income, which are known to be factors of health care engagement. Their influence on adherence may have been underestimated. Third, our sample size (N=108) was adequate for the primary analysis; it may be underpowered to detect predictors with more subtle effects. Finally, although we performed external validation, both cohorts were recruited from a single hospital. This shared clinical and demographic context limits the generalizability of our nomogram. Studies are needed to validate our model in multicenter or community-based cohorts to confirm its broader applicability.

### Conclusions

In conclusion, this study is the first to develop and validate a predictive model for acupuncture treatment adherence in patients with AD, offering a novel, evidence-based tool for both clinical research and practice. For clinical research, this model provides a method to stratify enrollment or identify participants who may require enhanced adherence support, thereby reducing bias and improving the integrity of future trials. In clinical practice, the nomogram enables a shift from reactive problem-solving to proactive adherence management. By prospectively identifying patients at high risk, clinicians can address specific barriers and implement targeted strategies to improve adherence and, ultimately, enhance therapeutic outcomes.

## Supplementary material

10.2196/82787 Multimedia Appendix 1 .Supplementary analyses supporting the development and validation of the adherence prediction model.

## Abbreviations

AD: Alzheimer disease
AUC: area under the receiver operating characteristic curve
CACMS: China Academy of Chinese Medical Sciences
OR: odds ratio
PDC: proportion of days covered
RCT: randomized controlled trial
ROC: receiver operating characteristic
SHAP: Shapley Additive Explanations

## References

[1] Tatulian SA (2022). Challenges and hopes for Alzheimer’s disease. Drug Discov Today.

[2] Dong X qing, Li X ying, Kong X he (2020). Analysis of clinical application patterns in acupuncture-moxibustion treatment of Alzheimer disease. J Acupunct Tuina Sci.

[3] Xia KP, Pang J, Li SL, Zhang M, Li HL, Wang YJ (2020). Effect of electroacupuncture at governor vessel on learning-memory ability and serum level of APP, Aβ_1-42_ in patients with Alzheimer’s disease. Zhongguo Zhen Jiu.

[4] Ouyang Q, Mu YY (2000). Clinical observation of electroacupuncture combined with perphenazine in treating psychiatric symptoms of Alzheimer’s disease. Shanghai J Acupunct Moxibustion.

[5] Lin L, Ma X, Wang G, Wang HZ, Wang ZQ, Wang ZW (2024). Chinese guidelines for early prevention of Alzheimer’s disease (2024). Chin J Alzheimer Dis Relat Disord.

[6] Wang YY, Yu SF, Xue HY, Li Y, Zhao C, Jin YH (2020). Effectiveness and safety of acupuncture for the treatment of Alzheimer’s disease: a systematic review and meta-analysis. Front Aging Neurosci.

[7] Ke C, Shan S, Yu J, Wei X, Pan J, Zhang W (2024). Acupuncture for patients with Alzheimer’s disease: an evidence map of randomized controlled trials, systematic reviews, and meta-analysis. J Alzheimers Dis.

[8] Olchanski N, Daly AT, Zhu Y (2023). Alzheimer’s disease medication use and adherence patterns by race and ethnicity. Alzheimers Dement.

[9] Xiong S, Wu J, Li M, Lu K (2021). PDG38 association between satisfaction with quality of care and ANTI-dementia medication adherence among elderly adults with Alzheimer’s disease and related dementias. Value Health.

[10] Wu XW, Yang HB, Yuan R, Long EW, Tong RS (2020). Predictive models of medication non-adherence risks of patients with T2D based on multiple machine learning algorithms. BMJ Open Diabetes Res Care.

[11] Koesmahargyo V, Abbas A, Zhang L (2020). Accuracy of machine learning-based prediction of medication adherence in clinical research. Psychiatry Res.

[12] Nagpal TS, Mottola MF, Barakat R, Prapavessis H (2021). Adherence is a key factor for interpreting the results of exercise interventions. Physiotherapy.

[13] Wang XL, Hu HQ, Wen Q, Li N (2021). Considerations and suggestions on influencing factors of compliance in clinical trial of acupuncture and moxibustion: experience in case study of knee osteoarthritis treated with acupuncture. Zhongguo Zhen Jiu.

[14] Duan HL, Du ZJ, Zhang WR, Dong XF (2018). Influencing factors of acupuncture adherence based on literature review. World's Latest Medical Information Digest.

[15] Yu MK, Hu RX, Wen LZ, Li X, Zhao ZY (2019). Characteristics of the methodological studies on patient compliance in clinical trials in China. Chin J EvidBased Med.

[16] Wei J, Liu HL, Wang LC, Wang LP, Liu ZS, Yi Y (2014). Discussion on improving compliance of participants in acupuncture-moxibustion clinical trial. Beijing J Tradit Chin Med.

[17] McKhann GM, Knopman DS, Chertkow H (2011). The diagnosis of dementia due to Alzheimer’s disease: recommendations from the National Institute on Aging-Alzheimer’s Association workgroups on diagnostic guidelines for Alzheimer’s disease. Alzheimers Dement.

[18] Cui Y, Zhang J, Wang Y (2025). Multivariate predictive model of the therapeutic effects of metoprolol in paediatric vasovagal syncope: a multi-centre study. EBioMedicine.

[19] Lundberg SM, Lee SI (2017). A unified approach to interpreting model predictions. https://proceedings.neurips.cc/paper_files/paper/2017/file/8a20a8621978632d76c43dfd28b67767-Paper.pdf.

[20] David W, Lemeshow S, Sturdivant RX (2013). Applied Logistic Regression.

[21] Wang YY, Liu Z, Wu Y (2016). Acupuncture for smoking cessation in Hong Kong: a prospective multicenter observational study. Evid Based Complement Alternat Med.

[22] White A, Cummings M, Barlas P (2008). Defining an adequate dose of acupuncture using a neurophysiological approach--a narrative review of the literature. Acupunct Med.

[23] Xu G, Lei H, Huang L (2022). The dose-effect association between acupuncture sessions and its effects on major depressive disorder: a meta-regression of randomized controlled trials. J Affect Disord.

[24] Zhang Y, Yang R, Zhang C, Han L (2025). Dose-effect relationship between the number of acupuncture sessions and efficacy for cervical vertigo: a meta-regression analysis based on randomized controlled trials. Zhongguo Zhen Jiu.

[25] Li J, Aulakh N, Culum I, Roberts AC (2024). Adherence to non-pharmacological interventions in Parkinson’s disease: a rapid evidence assessment of the literature. J Parkinsons Dis.

[26] Wang TS, Bai P (2019). Clinical study on 37 cases of periarthritis treated with different acupuncture frequencies. Jiangsu J Tradit Chin Med.

[27] Li Y (2013). Study on the time-effect relationship of acupuncture between needle retaining time and the interval time of acupuncture. Liaoning J Tradit Chin Med.

[28] (2020). Caregiving in the United States 2020. https://www.hrsa.gov/sites/default/files/hrsa/advisory-committees/nursing/reports/report-caregiving-us-2020.pdf.

[29] Schulz R, Eden J, Committee on Family Caregiving for Older Adults, Board on Health Care Services, Health and Medicine Division, National Academies of Sciences, Engineering, and Medicine (2016). Families Caring for an Aging America.

[30] Chen S, Cao Z, Nandi A (2024). The global macroeconomic burden of Alzheimer’s disease and other dementias: estimates and projections for 152 countries or territories. Lancet Glob Health.

[31] Xu P, Zhong QL (2016). Research progress on long-term care services for disabled elderly in community-based home settings. Chin J Gerontol.

[32] Ma WJ, Wang YH (2018). Influencing factors of informal home care time. Chin J Gerontol.

[33] Berge LI, Angeles RC, Gedde MH (2025). Burden and care time for dementia caregivers in the LIVE@Home.Path trial. Alzheimers Dement.

[34] Golics CJ, Basra MKA, Finlay AY, Salek S (2013). The impact of disease on family members: a critical aspect of medical care. J R Soc Med.

[35] Feng Y, Wu QH, Lin SB, Lin H, Huang XH, Liu BB (2022). Study on the correlation between caregiver burden, coping style, and acceptance behavior in primary caregivers of Alzheimer’s disease patients. Chin Gen Pract Nurs.

[36] Du X, Shen J, Wu YF (2015). Correlation between caregiving experience and fatigue among dementia caregivers in old age. Chin J Gerontol.

[37] Pinquart M, Sörensen S (2007). Correlates of physical health of informal caregivers: a meta-analysis. J Gerontol B Psychol Sci Soc Sci.

[38] Wenborn J, O’Keeffe AG, Mountain G (2021). Community occupational therapy for people with dementia and family carers (COTiD-UK) versus treatment as usual (Valuing Active Life in Dementia [VALID]) study: a single-blind, randomised controlled trial. PLoS Med.

[39] Lamb SE, Sheehan B, Atherton N (2018). Dementia And Physical Activity (DAPA) trial of moderate to high intensity exercise training for people with dementia: randomised controlled trial. BMJ.

[40] Lilly MB, Laporte A, Coyte PC (2007). Labor market work and home care’s unpaid caregivers: a systematic review of labor force participation rates, predictors of labor market withdrawal, and hours of work. Milbank Q.

[41] Liu JH, Wang XL, Li WL, Yang L, Wang JG (2021). Effect of synchronous education for children caregivers on hip function recovery after intertrochanteric fracture surgery in elderly patients. Clin Res.

[42] Liu YY, Zhang L (2022). Application of children caregivers’ synchronous education in postoperative rehabilitation of elderly patients undergoing heart valve replacement. Contemp Nurse.

[43] Coley N, Gallini A, Garès V, Gardette V, Andrieu S, ICTUS/DSA group (2015). A longitudinal study of transitions between informal and formal care in Alzheimer disease using multistate models in the European ICTUS cohort. J Am Med Dir Assoc.

[44] MacNeil-Vroomen JL, Thompson M, Leo-Summers L, Marottoli RA, Tai-Seale M, Allore HG (2020). Health-care use and cost for multimorbid persons with dementia in the National Health and Aging Trends Study. Alzheimers Dement.

[45] Balkrishnan R, Housman TS, Carroll C, Feldman SR, Fleischer AB (2003). Disease severity and associated family impact in childhood atopic dermatitis. Arch Dis Child.

[46] Zhang DY, Yang JM, Liu H (2018). Correlation between psychological resilience and caregiving burden among caregivers of patients with senile dementia. Chin Gen Pract Nurs.

