# Supplementary material for: Predictive Model of Acupuncture Adherence in Alzheimer Disease: Secondary Analysis of Randomized Controlled Trials
Source: JMIR Aging. 2026 Jan 21;9:e82787. doi: 10.2196/82787 (PMC12822864; doi:10.2196/82787)
Supplement: Multimedia Appendix 1 [file aging-v9-e82787-s001.docx]

| **Variable** | **VIF** | **Tolerance** |
| --- | --- | --- |
| Sex: Male | 1.480 | 0.676 |
| Age (years) | 1.551 | 0.645 |
| Disease Duration (months) | 1.653 | 0.605 |
| Severity: Moderate | 3.971 | 0.252 |
| Severity: Severe | 7.683 | 0.130 |
| Occupation Type: Physical Work | 1.757 | 0.569 |
| ADAS-Cog Score | 8.407 | 0.119 |
| BADL Score | 4.854 | 0.206 |
| IADL Score | 6.488 | 0.154 |
| BPSD Status: Yes | 1.496 | 0.668 |
| PHQ.9: Status Normal | 1.535 | 0.651 |
| Education Level: No Higher Education | 1.984 | 0.504 |
| Travel Time to the Hospital (min) | 1.542 | 0.649 |
| Recruitment Method: Multimedia | 6.153 | 0.163 |
| Recruitment Method: Nursing Home | 3.051 | 0.328 |
| Recruitment Method: Outpatient | 2.281 | 0.438 |
| History of Acupuncture Treatment: Yes | 5.269 | 0.190 |
| Number of Treatments in First Month | 1.380 | 0.725 |
| Caregiving Role: Part-time Caregiver | 1.637 | 0.611 |

**Supplementary Table 1. Results of the collinearity analysis conducted for the 16 variables included in this study.** VIF is variance inflation factor. ADAS-Cog is Alzheimer’s Disease Assessment Scale–Cognitive Subscale; BADL is Basic Activities of Daily Living; IADL is Instrumental Activities of Daily Living; BPSD is Behavioral and Psychological Symptoms of Dementia; PHQ-9 is Patient Health Questionnaire-9.

| Variables | Coefficient | SE | z-value | Wald | *P* value | OR | 95% CI |
| --- | --- | --- | --- | --- | --- | --- | --- |
| Disease Duration | −0.028 | 0.014 | −1.969 | 4643.270 | 0.049 | 0.972 | 0.944–0.999 |
| Number of Treatments in First Month | 1.120 | 0.362 | 3.091 | 71.541 | 0.002 | 3.064 | 1.676–7.010 |
| Caregiving Role: Part-time Caregiver | −1.683 | 0.733 | −2.295 | 0.064 | 0.022 | 0.186 | 0.039–0.724 |
| (Intercept) | −9.244 | 3.745 | −2.469 | 0.000 | 0.014 | 0.000 | 0.000–0.055 |

**Supplementary Table 2. Results of multivariable logistic regression analysis of factors associated with adherence to acupuncture treatment among patients with AD.** OR is odds ratio; CI is confidence interval; SE is standard error. The predictive model for adherence to acupuncture treatment among patients with Alzheimer’s disease was established as follows:$\log\left( P \right)=-9.244-0.028\times\text{Disease}\text{ }\text{Duration}+1.120\times\text{Number}\text{ }\text{of}\text{ }\text{Treatments}\text{ }\text{in}\text{ }\text{First}\text{ }\text{Month}-1.683\times\text{Caregiving}\text{ }\text{Role}$.

| **Variable** | **Total (n=66)** | **Good Adherence (n=43)** | **Poor Adherence (n=23)** | **Statistic** | ***P* value** |
| --- | --- | --- | --- | --- | --- |
| **Sex, n (%)** |  |  |  |  | 1 |
| Female | 16 (38.1%) | 11 (37.9%) | 5 (38.5%) |  |  |
| Male | 26 (61.9%) | 18 (62.1%) | 8 (61.5%) |  |  |
| **Age, years (mean ± SD)** | 72.8 ± 7.3 | 72.5 ± 7.0 | 73.5 ± 8.1 | −0.39 | 0.702 |
| **Disease duration, months (mean ± SD)** | 36.0 [24.2, 48.0] | 36.0 [25.0, 48.0] | 36.0 [24.0, 60.0] | 164 | 0.505 |
| **Disease severity, n (%)** |  |  |  |  | 0.201 |
| Mild | 19 (45.2%) | 13 (44.8%) | 6 (46.2%) |  |  |
| Moderate | 13 (31%) | 11 (37.9%) | 2 (15.4%) |  |  |
| Severe | 10 (23.8%) | 5 (17.2%) | 5 (38.5%) |  |  |
| **Education level, n (%)** |  |  |  | 1.44 | 0.231 |
| No higher education | 25 (59.5%) | 15 (51.7%) | 10 (76.9%) |  |  |
| Higher education | 17 (40.5%) | 14 (48.3%) | 3 (23.1%) |  |  |
| **Occupation, n (%)** |  |  |  | 0 | 1 |
| Manual work | 17 (40.5%) | 12 (41.4%) | 5 (38.5%) |  |  |
| Physical Work | 25 (59.5%) | 17 (58.6%) | 8 (61.5%) |  |  |
| **MMSE, median (mean ± SD)** | 17.2 ± 7.7 | 17.9 ± 7.5 | 15.5 ± 8.3 | 0.93 | 0.36 |
| **ADAS-Cog, median (IQR)** | 28.5 [15.0, 39.0] | 28.0 [15.0, 35.0] | 33.0 [14.0, 44.0] | 178 | 0.785 |
| **BADL, median (IQR)** | 10.0 [8.0, 12.0] | 10.0 [8.0, 12.0] | 10.0 [8.0, 12.0] | 207 | 0.617 |
| **IADL, median (IQR)** | 23.5 [16.2, 30.5] | 24.0 [17.0, 29.0] | 23.0 [13.0, 31.0] | 214 | 0.496 |
| **BPSD, n (%)** |  |  |  |  | 1 |
| Present | 3 (7.1%) | 2 (6.9%) | 1 (7.7%) |  |  |
| Absent | 39 (92.9%) | 27 (93.1%) | 12 (92.3%) |  |  |
| **PHQ-9, n (%)** |  |  |  |  | 0.019 |
| Depressive symptoms | 11 (26.2%) | 4 (13.8%) | 7 (53.8%) |  |  |
| Normal | 31 (73.8%) | 25 (86.2%) | 6 (46.2%) |  |  |
| **Time to travel to hospital, min (median [IQR])** | 120.0 [60.0, 180.0] | 120.0 [60.0, 180.0] | 180.0 [120.0, 180.0] | 128.5 | 0.094 |
| **First-month treatment sessions, median (IQR)** | 10.5 [9.0, 12.0] | 11.0 [9.0, 12.0] | 9.0 [2.0, 9.0] | 303.5 | 0.001 |
| **Caregiving role, n (%)** |  |  |  |  | 0.531 |
| Full-time caregiver | 24 (57.1%) | 18 (62.1%) | 6 (46.2%) | 0.39 |  |
| Part-time caregiver | 18 (42.9%) | 11 (37.9%) | 7 (53.8%) | 0.39 |  |
| **History of acupuncture, n (%)** |  |  |  |  | 0.453 |
| No | 31 (73.8%) | 20 (69%) | 11 (84.6%) |  |  |
| Yes | 11 (26.2%) | 9 (31%) | 2 (15.4%) |  |  |
| **Recruitment method, n (%)** |  |  |  |  | 1 |
| Multimedia | 30 (71.4%) | 20 (69%) | 10 (76.9%) |  |  |
| Study team referral | 8 (19%) | 6 (20.7%) | 2 (15.4%) |  |  |
| Outpatient clinic | 4 (9.5%) | 3 (10.3%) | 1 (7.7%) |  |  |

**Supplementary Table 3. Comparison of baseline characteristics between patients with good and poor adherence in the validation cohort.** Values are presented as mean (SD), median (interquartile range), or counts (%), as appropriate. *P*-values for testing differences between patients with good and poor adherence to acupuncture treatment were derived from independent-samples t-tests, Mann–Whitney U tests and χ² tests or Fisher’s exact tests. MMSE is Mini-Mental State Examination; ADAS-Cog is Alzheimer’s Disease Assessment Scale–Cognitive Subscale; BADL is Basic Activities of Daily Living; IADL is Instrumental Activities of Daily Living; BPSD is Behavioral and Psychological Symptoms of Dementia; PHQ-9 is Patient Health Questionnaire-9.

| Adverse Event | Development Cohort | Validation Cohort |
| --- | --- | --- |
| Unrelated to treatment |  |  |
| Ocular discomfort | 1 | 0 |
| Knee Injury | 1 | 0 |
| Fall | 4 | 2 |
| Gait Instability | 1 | 0 |
| Epigastric Discomfort | 1 | 2 |
| Fatigue | 2 | 1 |
| Diarrhea | 1 | 1 |
| COVID-19 | 4 | 3 |
| Common Cold | 4 | 2 |
| Total | 19 | 11 |

**Supplementary Table 4. Adverse events unrelated to treatment of development cohort and validation cohort.**


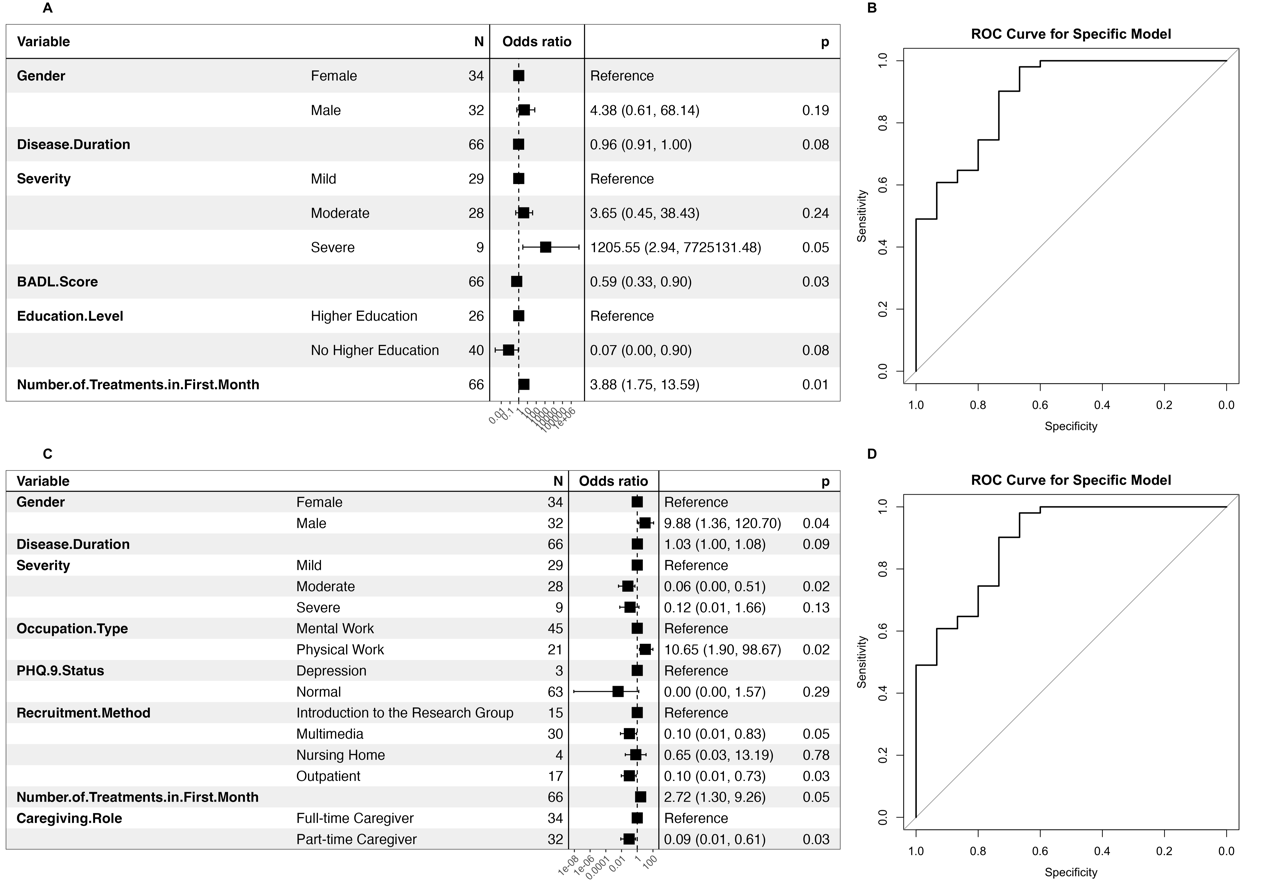


**Supplementary Figure 1. Sensitivity analyses of factors influencing adherence to acupuncture treatment among patients with Alzheimer’s disease under alternative adherence thresholds.** (A) Forest plot based on multivariable logistic regression using a 70% adherence threshold. (B) ROC curve for the 70% adherence threshold (AUC = 0.892). (C) Forest plot based on multivariable logistic regression using a 90% adherence threshold. (D) ROC curve for the 90% adherence threshold (AUC = 0.860).
